# Largely deformable torsional soft morphing actuator created by twisted shape memory alloy wire and its application to a soft morphing wing

**DOI:** 10.1038/s41598-023-44936-4

**Published:** 2023-10-17

**Authors:** Su-Yeon Lee, Gil-Yong Lee

**Affiliations:** 1 Department of Aeronautics, Mechanical and Electronic Convergence Engineering, Kumoh National Institute of Technology, Gumi, Gyeongbuk 39177 Republic of Korea; 2 Department of Mechanical Engineering, Kumoh National Institute of Technology, Gumi, Gyeongbuk 39177 Republic of Korea

**Keywords:** Aerospace engineering, Mechanical engineering

## Abstract

We present a new type of torsional soft morphing actuator designed and fabricated by twisted shape memory alloy (SMA) wires embedded in polydimethylsiloxane matrix. The design and fabrication process of the proposed soft morphing actuator are presented with investigations of its working mechanism. Actuation performance was evaluated with respect to the temporal response, the maximum torsional deformation under an applied electric current, and various design parameters including the twist direction, wire diameter, helical pitch of the SMA wire, and the actuator’s thickness and length. We demonstrate potential applications of the proposed soft morphing actuator as a soft morphing wing and airfoil. The proposed actuator will aid in the development of soft actuators, soft robotics, and other relevant scientific and engineering applications.

## Introduction

Shape memory alloy (SMA) is a material that can be restored to its undeformed shape with heat application following deformation under a low-temperature conditions^1–3^. This temperature-induced phase transformation is referred to as the shape memory effect. SMAs are lightweight, low in volume, and capable of quick response at small scale^3–5^; as such, they are well suited for miniaturization due to their relatively high power density^3–5^. Additionally, SMAs are easily actuated by resistive Joule heating, and the voltage applied for Joule heating is much lower than that required for piezoelectric or electrostatic actuators^3–6^. Such characteristics and advantages of SMAs have led to their wide application in soft actuators^5,7–14^, soft robotics^9,12,15–23^, morphing structures^24–31^, and so on. Although many SMA-based soft actuators and morphing structures have been proposed, few applications have involved the use of twisted SMA wires. Because SMA shrinks by the temperature-induced phase transformation, most types of SMA-based actuators use a linear movement^7,8,10,12,14,16,17,29,30^. In contrast, torsional soft actuators have received limited attention and only few works have been reported; for examples, a soft actuator using a single torsional pre-strained SMA wire embedded polydimethylsiloxane (PDMS)^13^, a soft composite actuator capable of twisting motion where multiple SMA wires were connected diagonally across the corners of a PDMS plate^5^, and a similar torsional composite actuator based on the polymer scaffold structure^6^. Here, we present a new type of torsional soft morphing actuator designed and fabricated using twisted SMA wires embedded in a soft PDMS matrix. The SMA wires are twisted by a stepper motor; the rotation direction of the stepper motor varies the twist direction of the SMA wires. The resulting twisted SMA wires are then folded and molded into the PDMS matrix. The demolded specimen generates torsional deformation against the twist direction of the SMA wire by application of an electric current. We investigated the torsional deformation of the proposed soft actuator with respect to various design parameters, including the twist direction, diameter and helical pitch of the SMA wire, and the thickness and length of the actuator. The actuation performances of the torsional soft morphing actuator prototypes were evaluated in terms of their temporal response and maximum achievable torsional deformation under various applied electric currents. Furthermore, the performance of our actuator prototype was demonstrated by soft morphing wings and an airfoil. Two morphed shapes of the soft morphing wings were demonstrated by combining different twist directions of the embedded SMA wires. With the soft morphing airfoil prototype, we were able to control the angle of attack through actuation of the embedded SMA wires. Additional design considerations for the proposed soft actuator were presented and discussed to emphasize its potential benefits for versatile applications. The results show that the proposed torsional soft morphing actuator is capable of producing large torsional deformation in effective and reliable manner, and it has enough potential to advance the field of soft robotics and morphing structure applications.

## Methods

### Design and fabrication of the torsional soft morphing actuators

**Figure 1:**
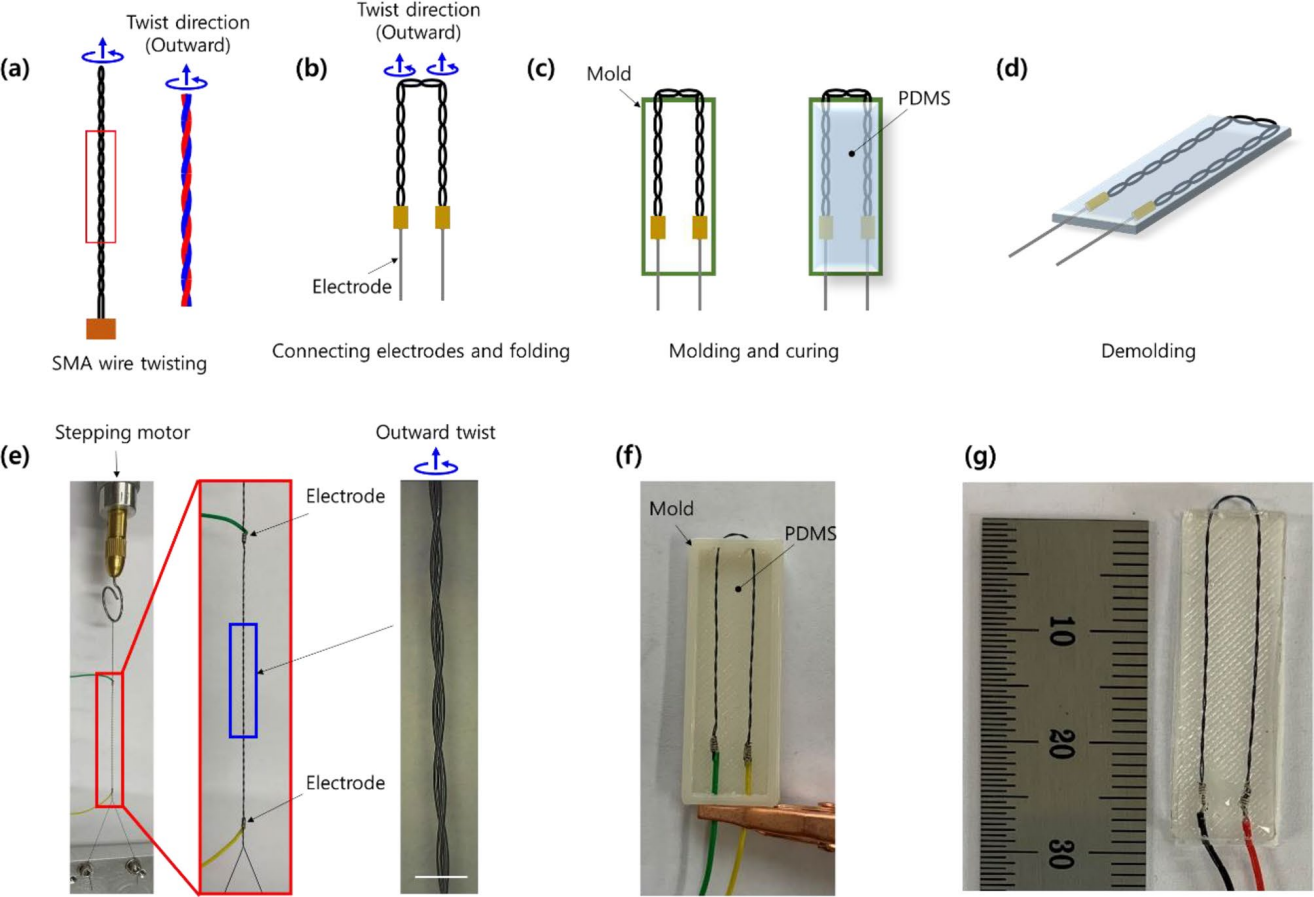
(**a**–**d**) Schematic illustrations of the fabrication processes of the twisted shape memory alloy (SMA) wire and the torsional soft morphing actuator. (**e**) Picture taken during the SMA wire twisting process by a stepper motor with a magnified image of the twisted SMA wire. Pictures of (**f**) the molded specimen and (**g**) a representative soft actuator specimen. Scale bar is 1 mm in the inset in (**e**).

Figure 1a–d schematically shows the fabrication processes of the proposed torsional soft morphing actuators. Two strands of the SMA wire were twisted using a stepper motor following the same procedure reported in our earlier study^32^. In the SMA wire-twisting process, two variations of the twist direction are possible. We denote the two directions as “inward” and “outward” twists along the twist axis (rotating axis of the stepper motor); the outward twist is depicted in Fig. 1a. The twisted SMA wire was connected to electrodes and folded as shown in Fig. 1b. The twist direction of the folded SMA wire is denoted using the same notation (inward or outward) with respect to the twist direction of the unfolded SMA wire by considering the twist axis. The twisted and folded SMA wire was then molded into the PDMS and cured (Fig. 1c). The final actuator specimen was prepared after demolding (Fig. 1d). Figure 1e shows the twisting process of the SMA wire using the stepper motor. The electrodes shown in Fig. 1e were prepared after finishing the twist of the SMA wire by wrapping conductive wires surrounding it. The magnified image of the twisted SMA wire taken by an optical microscope is included in the inset of Fig. 1e. Figure 1f shows a photo of the molded specimen. A representative actuator specimen is presented in Fig. 1g.

### Actuation mechanism of the torsional soft morphing actuators

**Figure 2:**
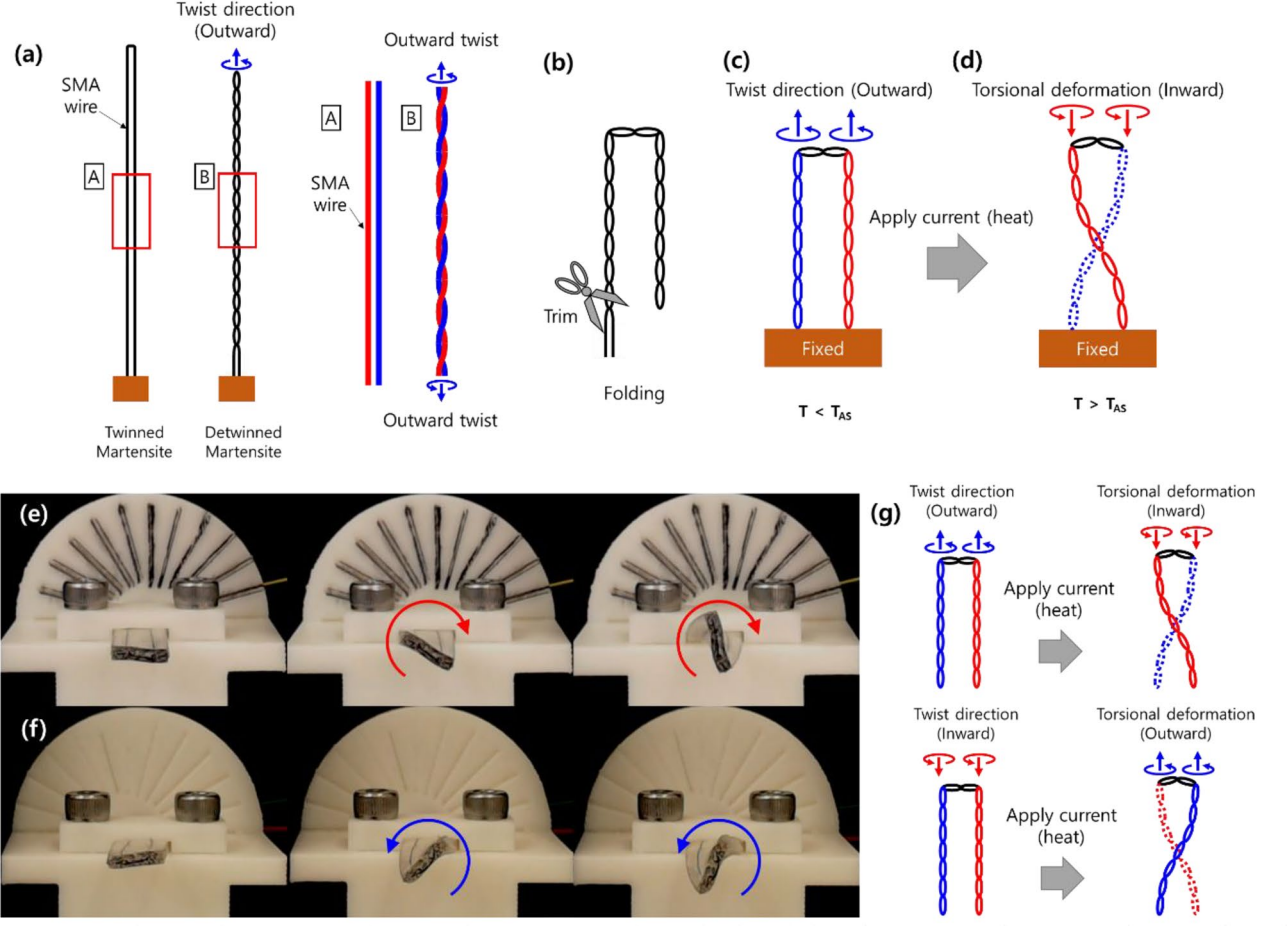
Schematic illustrations of (**a**) twisted and (**b**) folded SMA wire. (**c**, **d**) Torsional deformation is generated by applying heat to the twisted SMA wire. Snapshots capture the torsional deformations of the soft actuators fabricated by embedding (**e**) the outward twisted SMA wire and (**f**) the inward twisted SMA wire in a polydimethylsiloxane (PDMS) matrix, with (**g**) schematic diagrams showing the twist directions of the SMA wires and the resulting torsional deformation directions.

Figure 2a–d shows a schematic diagram of how torsional deformation is generated by the proposed twisted SMA wire. As the SMA wire is twisted (Fig. 2a), its crystal structure transforms from the twinned martensite to the detwinned martensite configuration. The detwinned phase is dominated by the torsional load applied to the SMA wire. Figure 2a shows the outward twist case. The inward and outward twist directions vary the direction of the torsional load for detwinning. Once the twisted SMA wire is trimmed and folded, its two free ends are fixed (Fig. 2b,c). Considering the twist axis of the SMA wire, the twist direction of the folded SMA wire remains the same as the unfolded one, as depicted in Fig. 2c (the outward twist case is shown as an example). By application of an electric current to the twisted SMA wire to create a temperature-induced phase transformation (from detwinned martensite to austenite), torsional moments are generated along the twist axis of the SMA wire against the twist direction resulting in torsional deformation (Fig. 2d). Supporting Movie S1 captures the corresponding torsional deformations of the inward and outward twisted SMA wires (without PDMS); 700 mA of step current was applied. By embedding the twisted SMA wire within a matrix (PDMS in our case), the entire structure exhibited torsional deformation. Figure 2e,f shows the resulting torsional deformation of the proposed soft actuators fabricated by embedding outward and inward twisted SMA wires in the PDMS, respectively, along with schematic illustrations of the corresponding motions of the twisted SMA wires in Fig. 2g; the step current was 700 mA.

### Actuator specimens and experimental setup

**Figure 3:**
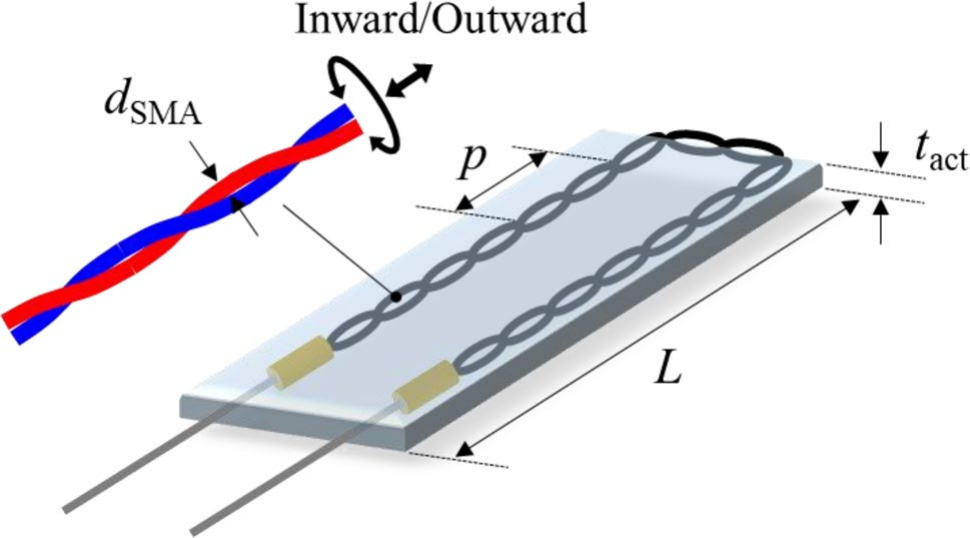
Variations in the design parameters of the torsional soft morphing actuators.

The actuation performance of the proposed soft actuator was investigated in terms of the temporal response and the maximum torsional deformation for various design parameters, including the twist direction, diameter and helical pitch of the SMA wire, and the thickness and length of the actuator specimen. Figure 3 shows a schematic representation of the design parameter variations.

Table 1 provides detailed information on the design parameters with their corresponding values for the prepared specimens used in the experiments. Six actuator specimen types, denoted as (S_1_)_out_, (S_1_)_in_, (S_2_)_out_, (S_3_)_out_, (S_4_)_out_, and (S_5_)_out_, were prepared. The subscript out (in) represents the outward (inward) twist direction of the twisted SMA wire in the soft actuator. Specimens (S_1_)_out_ and (S_1_)_in_ were fabricated by changing only the twist direction, while the other design parameters remained the same. Specimen (S_2_)_out_ had a longer helical pitch compared to (S_1_)_out_; the SMA wire twist direction, diameter, and the actuator thickness and length, were the same as those used in (S_1_)_out_. For specimen (S_3_)_out_, we used thinner SMA wire (100 µm); the other parameters remained the same as with (S_1_)_out_. Specimens (S_4_)_out_ and (S_5_)_out_ were designed to be thicker and longer, respectively, compared to specimen (S_1_)_out_ with the same SMA wire twist direction, diameter, and pitch.

**Table 1:** Design parameters of the torsional soft morphing actuator specimens used for the experiments.

	*d*_SMA_ (mm)	*p* (mm)	*t*_*act*_ (mm)	*L* (mm)	Twist direction (inward/outward)
(S_1_)_out_	150	3	2	20	Outward
(S_1_)_in_	150	3	2	20	Inward
(S_2_)_out_	150	6	2	20	Outward
(S_3_)_out_	100	3	2	20	Outward
(S_4_)_out_	150	3	4	20	Outward
(S_5_)_out_	150	3	2	40	Outward

**Figure 4:**
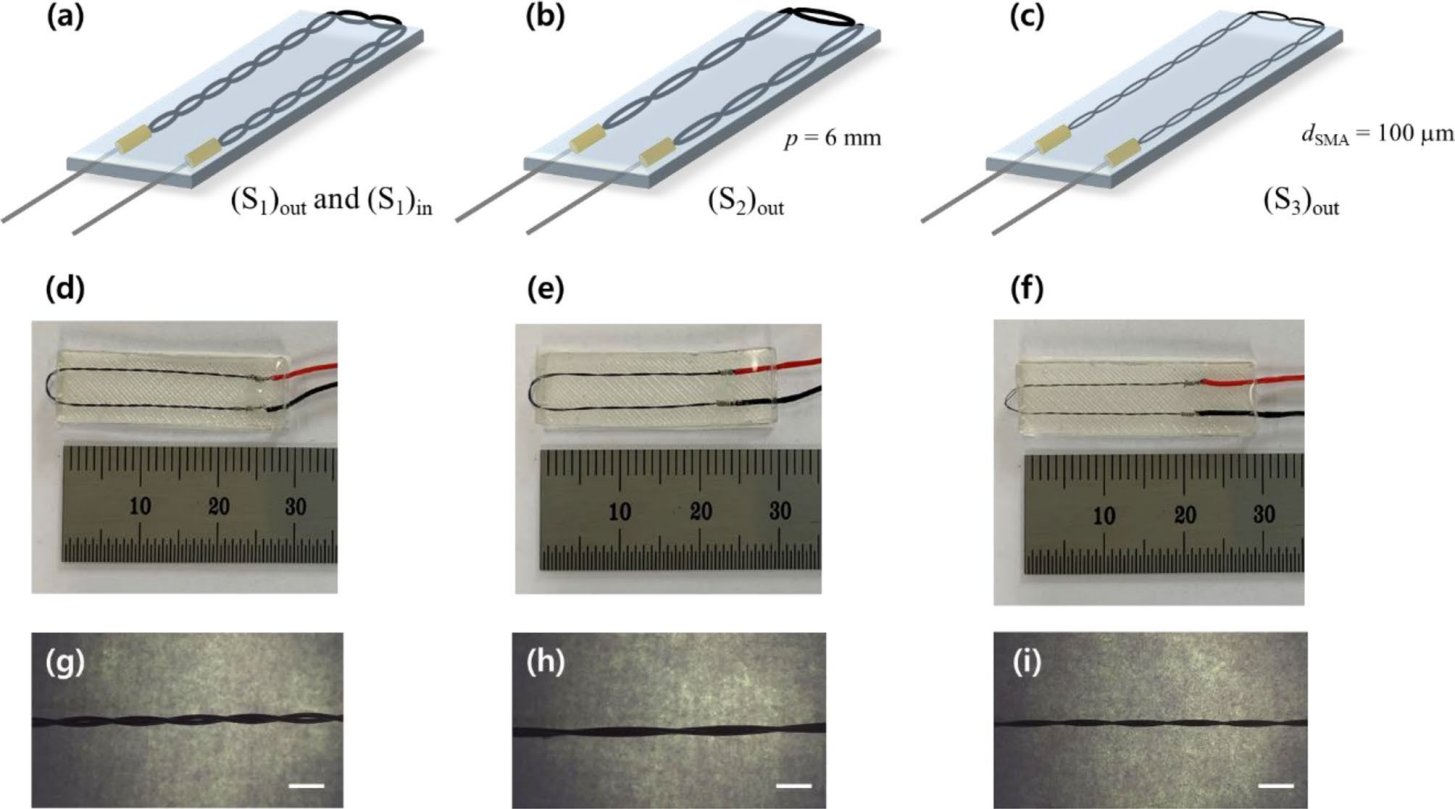
(**a**–**c**) Schematic diagrams and (**d**–**f**) photos of soft actuator specimens (S_1_)_out_, (S_1_)_in_, (S_2_)_out_, and (S_3_)_out_. (**g**–**i**) Optical microscope images capture the helical pitch and diameter of the twisted SMA wires within the soft actuator. Scale bars are 1 mm in (**g**–**i**).

**Figure 5:**
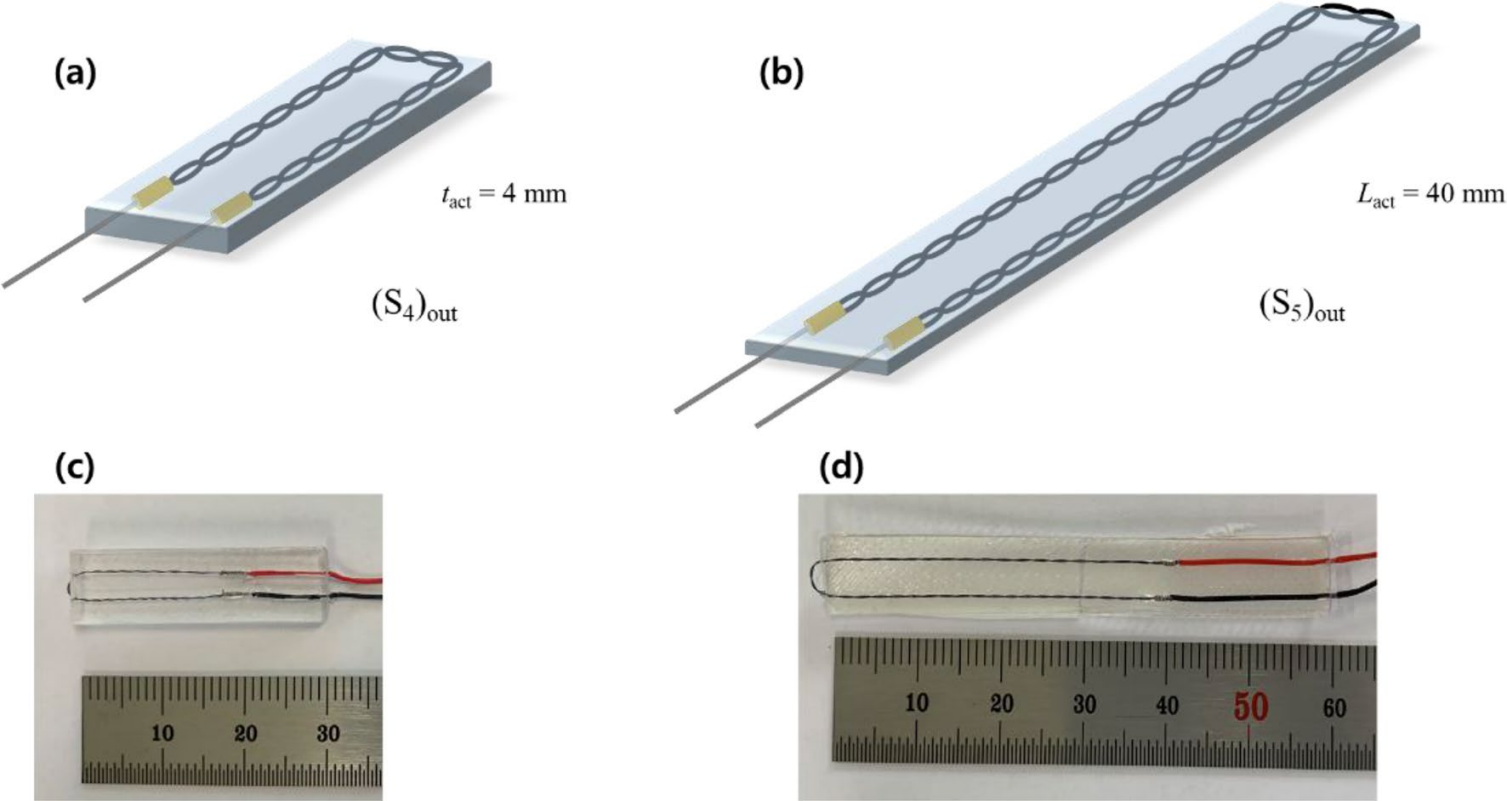
(**a**, **b**) Schematic diagrams and (**c**, **d**) photos of soft actuator specimens (S_4_)_out_ and (S_5_)_out_.

Figures 4 and 5 show representative soft actuator specimens fabricated and prepared following the described procedures, with the noted variations in the design parameters. Figure 4g–i are optical microscope images of the twisted SMA wires in specimens (S1)_out_, (S2)_out_, and (S3)_out_, respectively, for comparison of their helical pitch and diameter.

**Figure 6:**
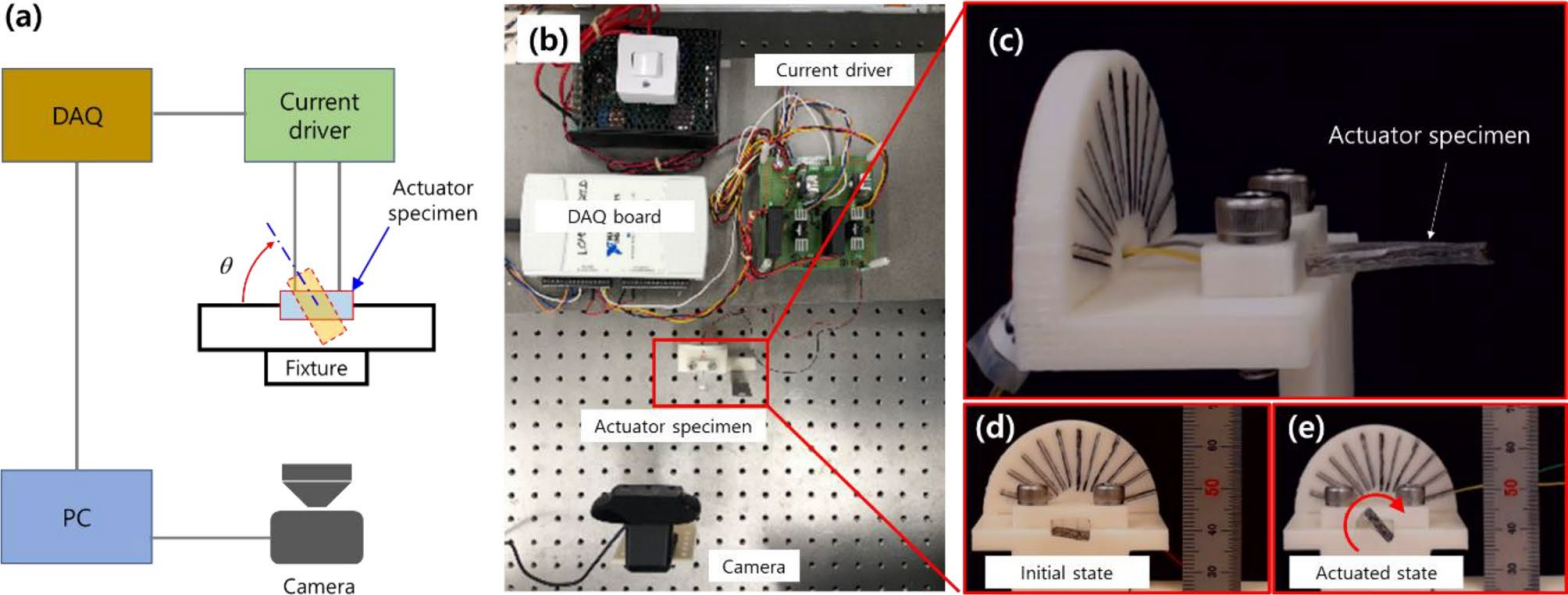
(**a**) Schematic diagram and (**b**) photo of the experimental setup. (**c**) Magnified image captured near the actuator specimen. (**d**) The soft actuator is in the initial state (before current is applied). (**e**) Torsional deformation from current application.

Our experimental setup consisted of a camera, a data acquisition (DAQ NI-USB6211) board, a custom-made current driver, and a specimen fixture, along with a personal computer, as shown in Fig. 6.

## Results and discussions

### Performance evaluations of the torsional soft morphing actuators

**Figure 7:**
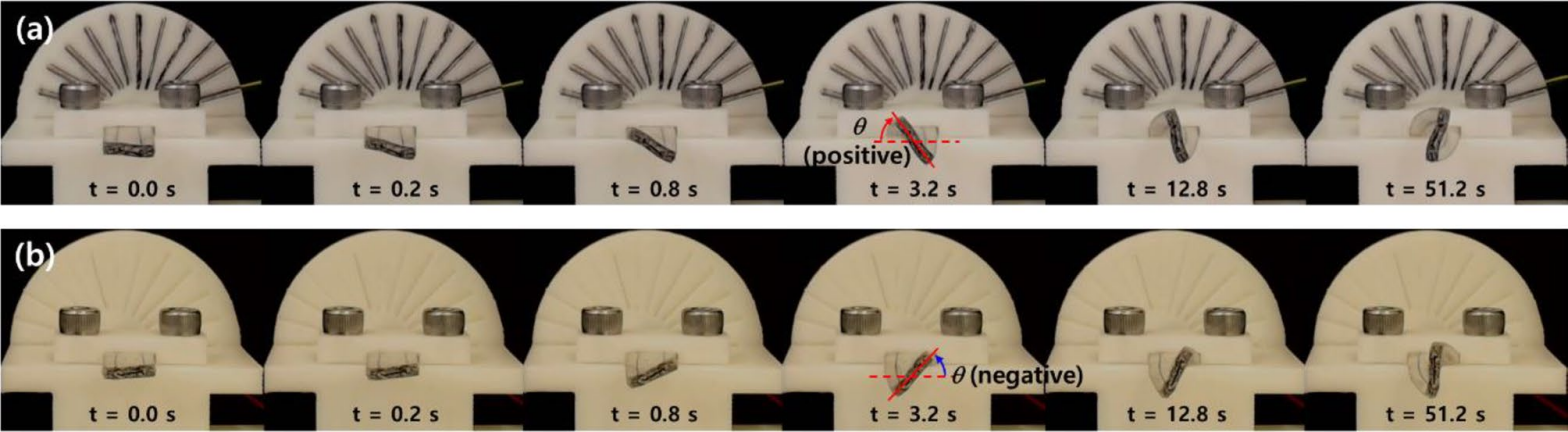
Temporal snapshots capture the torsional deformation of soft morphing actuators, (**a**) (S_1_)_out_ and (**b**) (S_1_)_in_, under a 700-mA step current input applied at *t* = 0 s.

**Figure 8:**
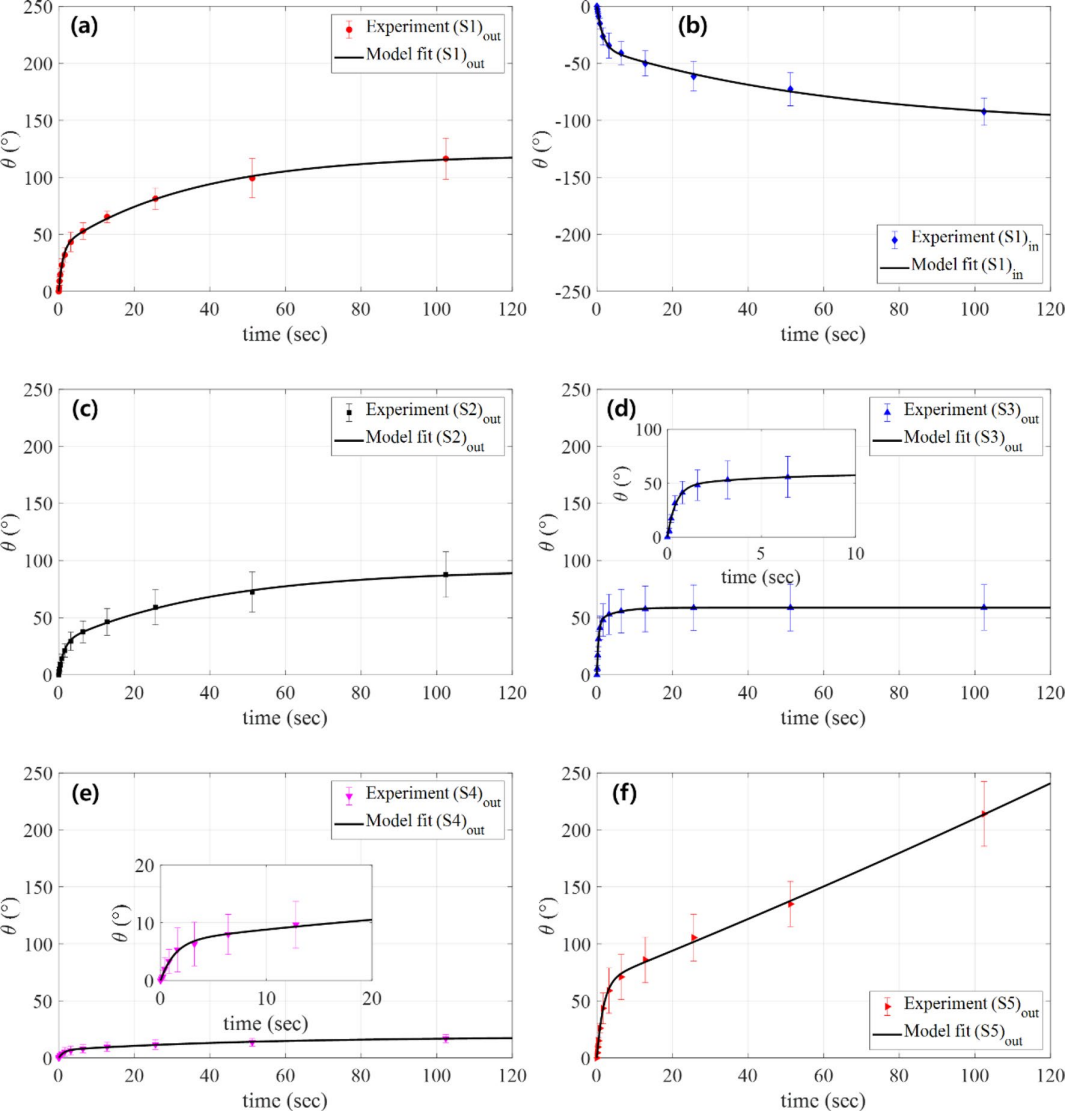
Temporal response plots for the torsional deformation of soft morphing actuators (**a**) (S_1_)_out_, (**b**) (S_1_)_in_, (**c**) (S_2_)_out_, (**d**) (S_3_)_out_, (**e**) (S_4_)_out_, and (**f**) (S_5_)_out_ under a 700-mA step current input applied at *t* = 0 s, along with the model fit.

To evaluate the torsional deformation of the soft actuator, the rotation angle of the actuator tip under application of a 700-mA step current to the actuator specimens (S_1_)_out_ and (S_1_)_in_ was measured and plotted with respect to time. Figure 7a,b show the temporal response of the torsional deformation of soft actuators (S_1_)_out_ and (S_1_)_in_, respectively. We set the positive direction of the torsional deformation as the rotating direction of the actuator tip with the outward twisted SMA wire (S_1_)_out_, as depicted in Fig. 7 (*i.e.*, the inward torsional deformation was defined as the positive direction). The measured tip rotation angles are plotted with respect to time in Fig. 8a,b. For specimens (S_1_)_out_ and (S_1_)_in_, the torsional deformation of the soft actuators approached + 57° and − 45.7° (average values) at *t* = 6.4 s with step current application, respectively, and the deformation increased to + 120.3° and − 97° at *t* = 102.4 s. The inward and outward torsional deformations showed nearly symmetric responses.

Assuming a linear time varying system, the equation of motion for the soft actuator can be expressed as Eq. (1). In Eq. (1), *J*_act_ is the mass moment of inertia, *C*_act_ is the equivalent damping coefficient, *K*_act_ is the equivalent spring constant of the soft actuator, and *τ* is the input torque.

1 $$J_{act} \frac{{d^{2} \theta }}{{dt^{2} }} + C_{act} \frac{d\theta }{{dt}} + K_{act} \theta = \tau = ai + b\frac{di}{{dt}}$$

Regarding the resistive heat generated in the SMA wire with application of a current to create the actuation force, it is plausible to assume that the input torque is proportional to the magnitude of the applied current and its time derivative. We included this assumption in Eq. (1), with coefficients *a* and *b* used for the forcing terms, and using a relationship such that the torque is proportional to the applied current *i* and its time derivative *di*/*dt*. The resulting transfer function *G*(*s*) = *θ*(*s*)/*I*(*s*) of the soft actuator is given by Eq. (2), where *θ*(*s*) is the Laplace transform of the tip rotation angle and *I*(*s*) is the Laplace transform of the input current. Because the proposed actuator did not show any oscillatory response in our experiments, we represent the transfer function with two real poles at *s* = –*α*, *s* = –*β* and one real zero at *s* = –*z* with gain *K*, as in Eq. (2).

2 $$G\left( s \right) = \frac{\theta \left( s \right)}{{I\left( s \right)}} = \frac{a + bs}{{J_{act} s^{2} + C_{act} s + K_{act} }} = \frac{{K\left( {s + z} \right)}}{{\left( {s + \alpha } \right)\left( {s + \beta } \right)}}$$

Then, the step response of the actuator tip rotation angle *θ*(*t*) is given by

3 $$\theta \left( t \right) = K\left( {\frac{z}{\alpha \beta } + \frac{z - \alpha }{{\alpha^{2} - \alpha \beta }}e^{ - \alpha t} + \frac{z - \beta }{{\beta^{2} - \alpha \beta }}e^{ - \beta t} } \right)$$

The experimental data were fitted to Eq. (3) by a nonlinear least square fit using MATLAB. The model fitted responses are plotted together in Fig. 8a,b. The soft actuator specimen (S_2_)_out_ fabricated with a longer helical pitch compared to (S_1_)_out_ and the specimen (S_3_)_out_ fabricated with a thinner SMA wire diameter compared to (S_1_)_out_ were evaluated in terms of their torsional deformation temporal response (*i.e.*, rotation angle of the actuator tip), along with their model fit, as shown in Fig. 8c,d. To further evaluate the performances of the torsional actuators for various structural parameters, we prepared soft actuator specimens with different actuator thicknesses and lengths, as denoted by (S_4_)_out_ and (S_5_)_out_ respectively, and plotted their temporal response in Fig. 8e,f along with the model fits. As presented in Fig. 8, the step responses of all soft actuators were well defined by Eq. (3). Table 2 provides the fitting coefficients and the *R*-squared values for the fitting curves. We also provide in-situ movies capturing the temporal responses of representative actuator specimens (S_1_)_out_, (S_1_)_in_, (S_2_)_out_, (S_3_)_out_, (S_4_)_out_, and (S_5_)_out_ under a 700-mA step current input in Supporting Movie S2.

**Table 2:** Fitting coefficients and *R*^2^ values of the model fits for the experimental data.

	*K*	*z*	*α*	*β*	*R*^2^
(S_1_)_out_	38.9767	0.0818	0.0284	0.9361	0.9989
(S_1_)_in_	− 24.4819	0.0437	0.0158	0.6431	0.9986
(S_2_)_out_	22.4585	0.0736	0.0245	0.7308	0.9990
(S_3_)_out_	109.1306	0.2413	0.1977	2.2578	0.9953
(S_4_)_out_	5.2646	0.0505	0.0194	0.7434	0.9972
(S_5_)_out_	40.0061	0.0180	0.5774	-0.0013	0.9991

**Figure 9:**
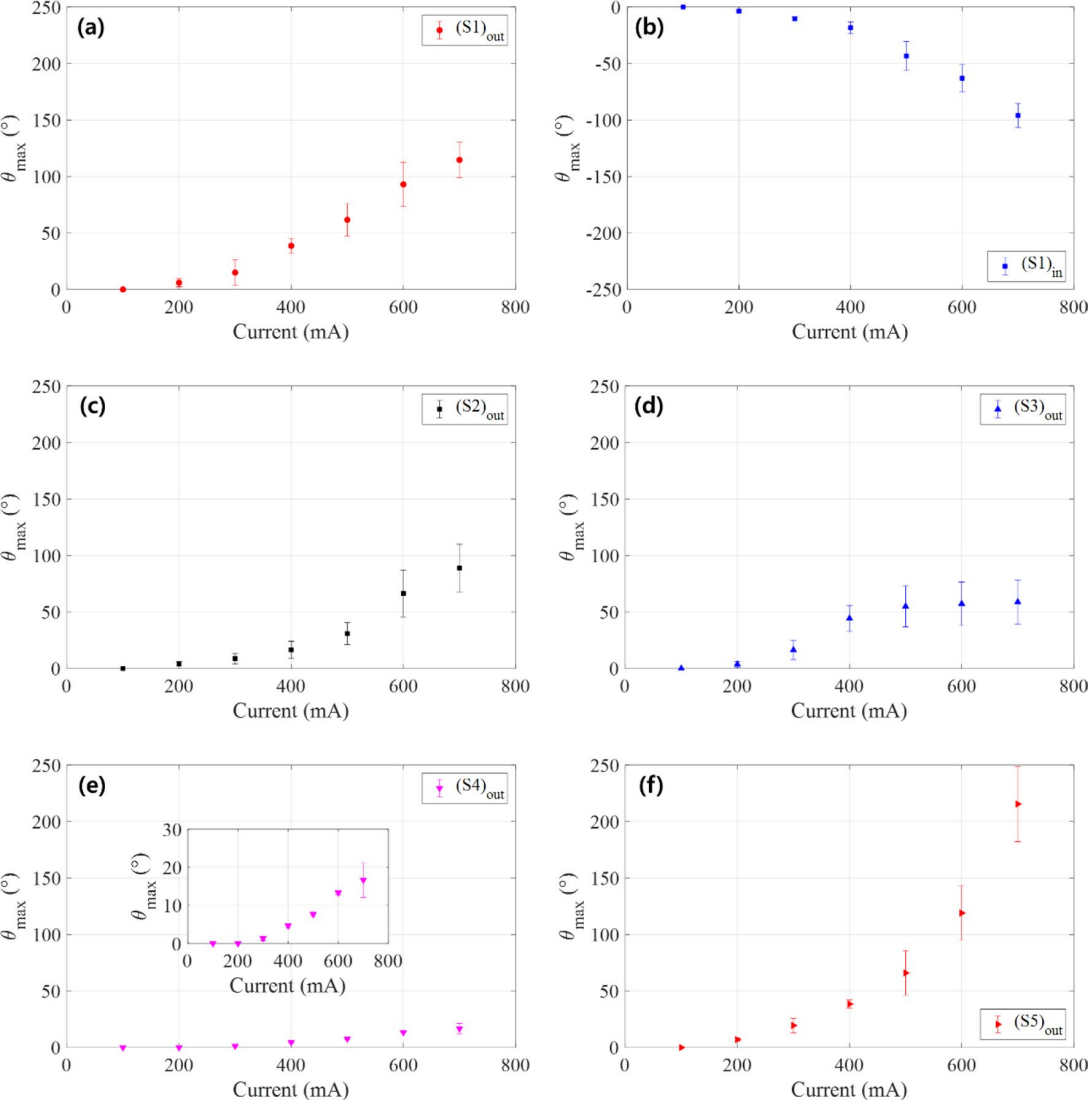
Plot of the maximum torsional deformation of soft morphing actuators (**a**) (S_1_)_out_, (**b**) (S_1_)_in_, (**c**) (S_2_)_out_, (**d**) (S_3_)_out_, (**e**) (S_4_)_out_, and (**f**) (S_5_)_out_, with respect to the amplitude of the step current input.

Referring to Fig. 8a–f, the torsional deformations for all actuator specimens reached a steady state at ~ 100 s after the step current was applied. We took the tip rotation angle at *t* = 102.4 s as the maximum torsional deformation of the soft actuator under the specified amplitude of the step current. Figure 9 shows the maximum torsional deformation (tip rotation angle measured at *t* = 102.4 s) with variations in the amplitude of the step current applied to soft actuator specimens (S_1_)_out_, (S_1_)_in_, (S_2_)_out_, (S_3_)_out_, (S_4_)_out_, and (S_5_)_out_.

As in Fig. 9, the tip angles for all specimens were very small for the 100-mA applied current, implying that the temperature-induced phase transformation of the SMA wire was not effectively initiated at this current input. The maximum torsional deformation (actuator tip angle) increased with the amplitude of the applied current from 200 to 700 mA. As the helical pitch increased, the SMA wire became less twisted. Thus, we expect reduced torsional deformation of the soft actuator using a longer helical pitch of the SMA wire. By comparing the temporal responses of specimens (S_2_)_out_ (Fig. 8c) and (S_1_)_out_ (Fig. 8a), and the maximum torsional deformation of specimens (S_2_)_out_ (Fig. 9c) and (S_1_)_out_ (Fig. 9a), we clearly observe such tendencies. The smaller the diameter of the SMA wire, the faster the response of the soft actuator; compare the temporal response of specimen (S_3_)_out_ (torsional actuator with the 100-µm-diameter SMA wire) in Fig. 8d to that of specimen (S_1_)_out_ (torsional actuator with a 150-µm-diameter SMA wire) in Fig. 8a. Additionally, as we used thinner SMA wire for the actuator, the maximum torsional deformation decreased; compare Fig. 9d to a. The reduced mass of the thinner SMA wire allowed it to absorb more heat compared to the thicker SMA wire under the same current input (resistive heat), resulting in faster initiation of the temperature-induced phase transformation. Additionally, the maximum deformation generated by the thinner SMA wire decreased because the actuation force of the SMA wire was reduced by its smaller mass.

As shown in Figs. 8e and 9e, as the actuator thickness increased, the torsional deformation decreased because the torsional area moment of inertia was larger. The torsional deformation of the longer soft actuator was remarkably enhanced, as shown in Figs. 8f and 9f. The torsional deformation of the soft actuator decreased (increased) as the thickness increased (as the length increased). These results are consistent with a simple estimation of the angle of twist for a torsional beam, *θ* = *τL*/*G*_act_*J*, where *θ* is the tip angle at the free end, *τ* is the applied torque, *L* is the length of the beam, *G*_act_ is the shear modulus, and *J* = *wt*_act_(*w*^2^ + *t*_act_^2^)/12 is the second polar moment of area (*w* is the width and *t*_act_ is the thickness of the rectangular cross-section of the soft actuator).

### Soft morphing wings

**Figure 10:**
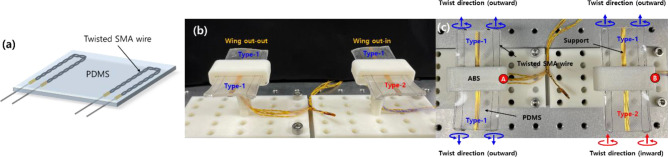
(**a**) Schematic diagram of the morphing wing composed of two twisted SMA wires. (**b**, **c**) Picture of the prototypes combined with the Type-1 and Type-2 wings. The red-circled markers “A” and “B” in (**c**) are denoted to indicate the front of the prototypes.

The proposed torsional actuator provides large torsional deformation under the application of an electric current to the twisted SMA wire. The rotation direction can be easily tuned by changing the twist direction. Integrating twisted SMA wires into a soft matrix such as PDMS allows for the creation of active soft morphing structures. As many types of soft morphing structures have been reported, to our knowledge, few if any have investigated morphing structures based on twisted SMA wires to date. In this regard, with the aim of providing a potential application of the proposed soft actuator, we designed and fabricated soft morphing wing prototypes that combined the twisted SMA wires with different twist directions into a single wing-like structure. Figure 10a shows a schematic diagram of the wing prototype design. Two twisted SMA wires were embedded into a flat PDMS matrix to create the morphing wing. Two combinations of twist directions of the composing SMA wires were considered in the wing design. The Type-1 wing used two embedded outward-twisted SMA wires, and the Type-2 wing used two embedded inward-twisted SMA wires. We combined Type-1 and Type-2 variations for each wing of the prototype, as presented in Fig. 10b,c. The first prototype [the one on the left in Fig. 10b,c] comprised two Type-1 wings; as such, this prototype was denoted as “Wing out-out.” The second prototype [the one on the right in Fig. 10b,c] was composed of one Type-1 left wing and one Type-2 right wing, denoted as “Wing out-in.” We depict the twist directions of the composing SMA wires within each prototype in Fig. 10b,c.

**Figure 11:**
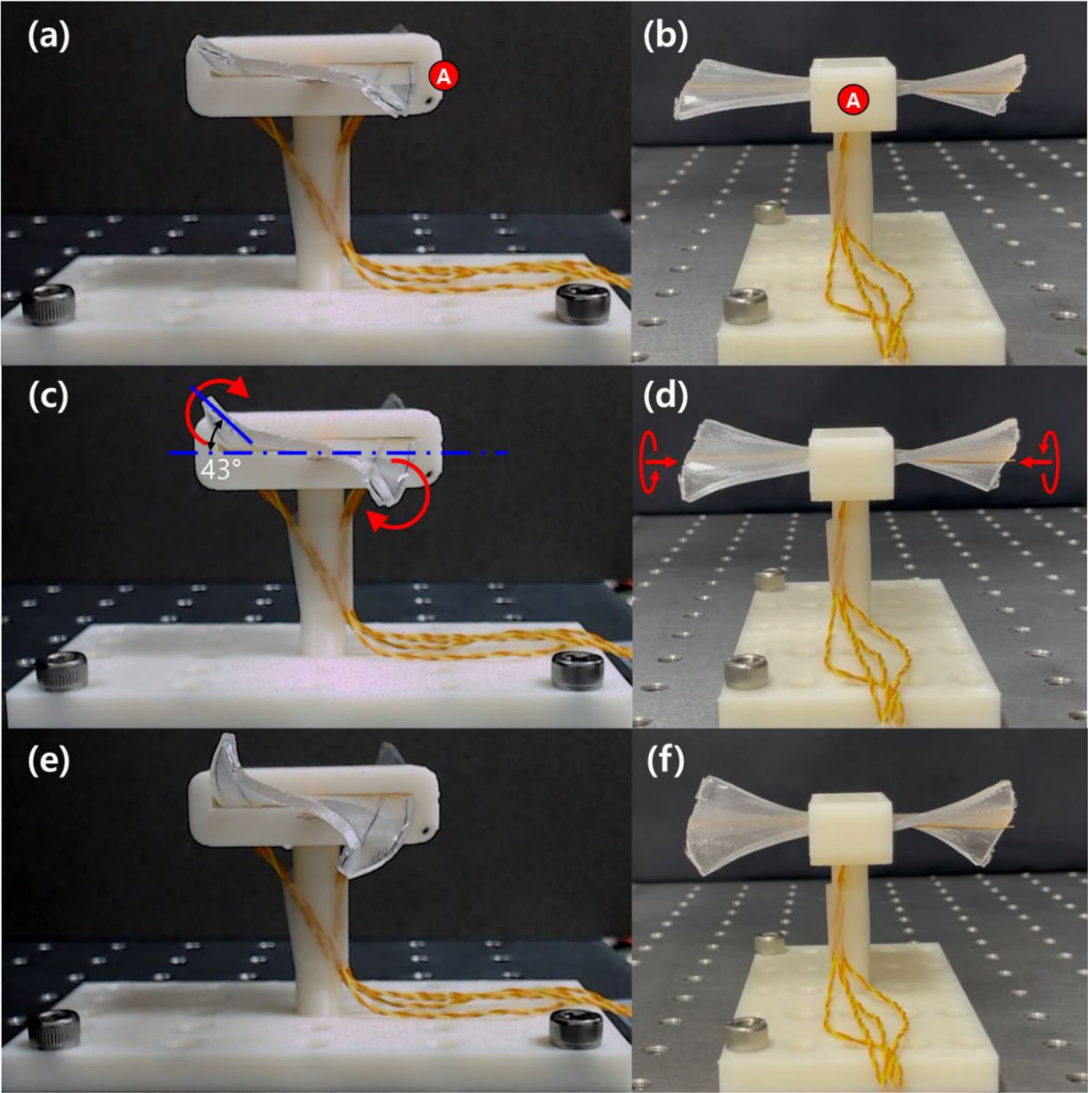
Morphed shapes of the soft morphing wing prototype (Wing out-out) taken at 10 s after a (**a**, **b**) 500-, (**c**, **d**) 600-, and (**e**, **f**) 700-mA step current was applied. The red-circled marker “A” indicates the front of the prototype and corresponds to the same marker depicted in Fig. 10c.

Figure 11 shows the morphed shapes of the first prototype (Wing out-out) on application of different currents (500–700 mA); the photos were taken 10 s after the current was applied. We provide captured images taken from the side (right wing) (Fig. 11a,c,e) and front (Fig. 11b,d,f) of the prototype. The red-circled marker “A” in Figs. 10c and 11a,b indicates the front of the prototype. Because we included the two twisted SMA wires near the front and rear ends of the morphing wing (Fig. 10a), the right wing of the prototype, composed of two outward-twisted SMA wires, generated torsional moments deforming the morphing wing to a side-lying “S” shape (Fig. 11a,c,e). As the applied current increased from 500 to 700 mA, the curvature of the morphed wing increased dramatically.

**Figure 12:**
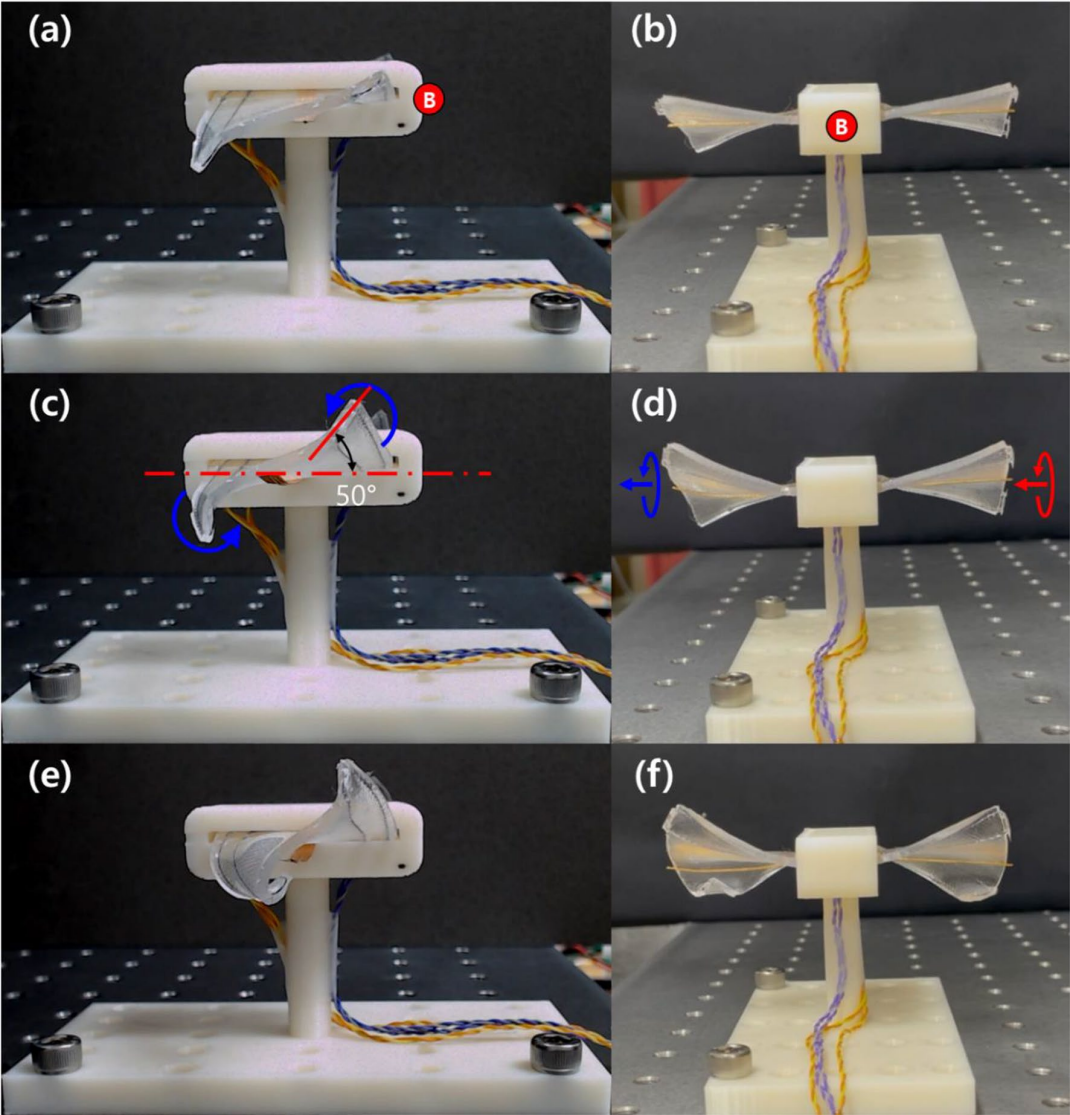
Morphed shapes of the soft morphing wing prototype (Wing out-in) taken 10 s after the application of a (**a**, **b**) 500 mA, (**c**, **d**) 600 mA, and (**e**, **f**) 700 mA step current. The red-circled marker “B” indicates the front of the prototype and corresponds to the same marker depicted in Fig. 10c.

The second prototype (Wing out-in) is shown with its morphed shapes under current inputs of 500–700 mA in Fig. 12 (the photos were taken 10 s after the current was applied). Figure 12a,c,e captures the side view (right wing) and Fig. 12b,d,f shows the front view of the prototype. The red-circled marker “B” in Figs. 10c and 12a,b indicates the front of the prototype. Because the right wing of the second prototype was constructed from two inward-twisted SMA wires, the direction of torsional deformation was flipped compared to the first prototype. Thus, the morphing wing deformed to a side-lying “Z” shape. We again observed an abrupt increase in wing deformation as the current increased from 500 to 700 mA.

Supporting Movies S3 and S4 provide movie clips of the two morphing wing prototypes presented in Figs. 11 and 12, actuated under various applied currents. When we take a look at each prototype from the front (indicated by red-circled markers A and B), the two wings of the first prototype exhibited an anti-symmetric configuration, while the two wings of the second prototype showed a symmetric configuration. The structural configuration can be tuned easily by combining different twist directions of the composing twisted SMA wires. Here we present two examples of the possible combinations.

### Soft morphing airfoils

**Figure 13:**
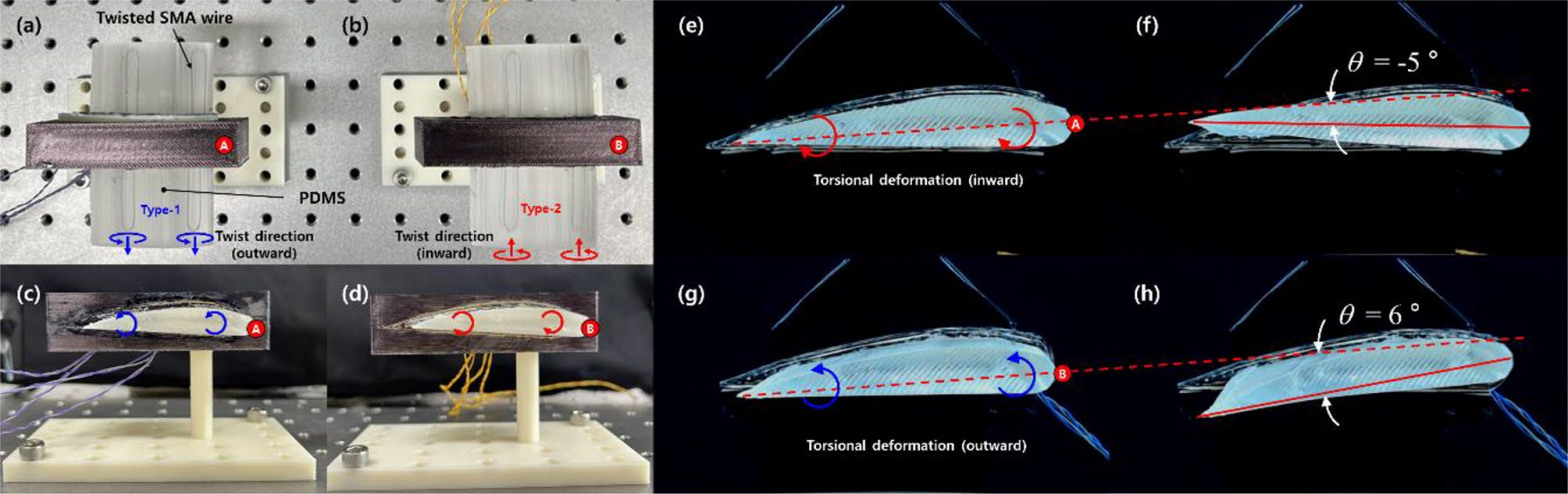
(**a**, **b**) Fabricated airplane-shape prototypes composed of soft morphing airfoils embedded with the twisted SMA wires. (**c**, **d**) Side view (right wing) of each prototype. Snapshots of the Type-1 airfoil taken at (**e**) the initial state and (**f**) the actuated state (700-mA current). Snapshots of the Type-2 airfoil taken at (**g**) the initial state and (**h**) the actuated state (700 mA current). The red-circled markers “A” and “B” indicate the front of each prototype.

We further extended the proposed concept to a soft morphing airfoil. Figure 13 presents small airplane-shaped prototypes composed of soft morphing airfoils embedded with two twisted SMA wires with different combinations of twist directions (Type-1 and Type-2). The twist directions of the right wings of each prototype are indicated in Fig. 13a–d. We applied 700 mA of step current input to the airfoils and took snapshots of the initial state and the actuated states 4 s after the step current was applied, as shown in Fig. 13e–h. The corresponding movie taken during the experiments is provided as Supporting Movie S5. As presented in Fig. 13e,f, the Type-1 airfoil composed of two outward twisted SMA wires generated inward torsional deformation, decreasing the angle of attack of the airfoil. The decrease in the angle of attack was approximately 5°, as shown in Fig. 13f. Figure 13g,h shows the increased angle of attack of 6° for the Type-2 airfoil composed of two inward twisted SMA wires, as indicated in Fig. 13h. The simple demonstrations presented herein prove the applicability of the proposed soft actuator for a new type of morphing wing and airfoil.

### Further design considerations for the torsional soft morphing actuators

**Figure 14:**
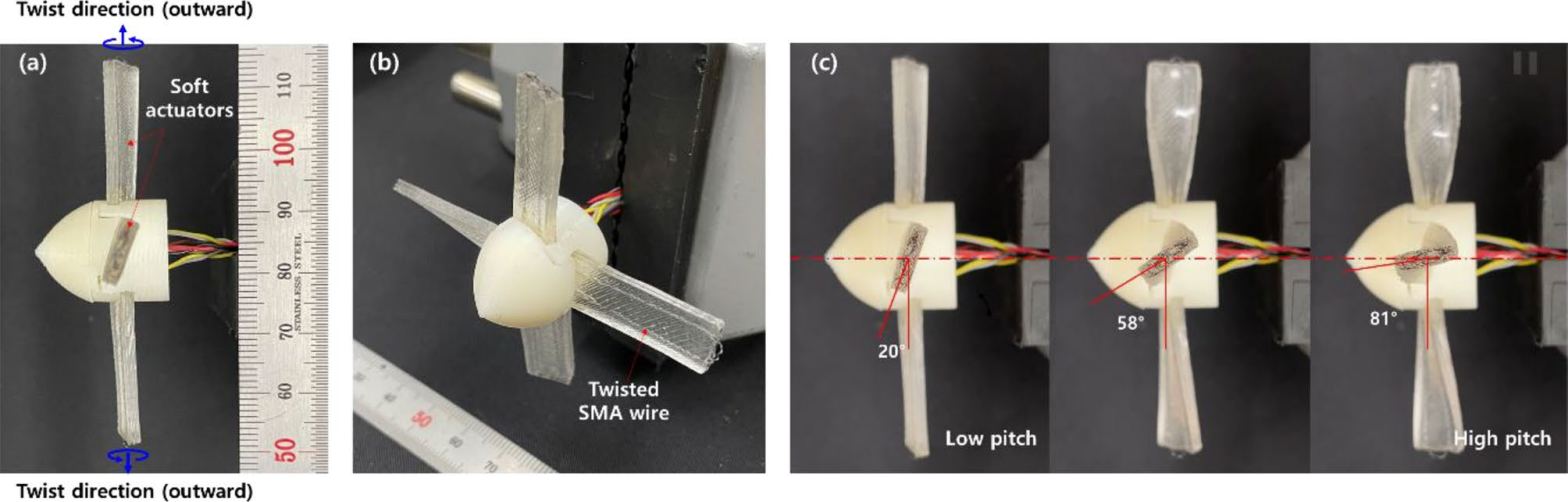
(**a**, **b**) Fabricated propeller prototype composed of soft morphing actuators embedded with the twisted SMA wires. (**c**) Snapshots show temporal responses of the propeller prototype upon the application of an electric current (1.5 A). Time duration between each snapshot in (**c**) was approximately 1 s.

We provide additional design considerations for the proposed torsional soft actuators with application examples to enhance their potential advantages. For example, the rotary motion generated by our soft actuator can be implemented for variable pitch control of a propeller. As presented in Fig. 14a,b, a simple propeller prototype was fabricated by configuring four soft actuators with the outward twisted SMA wires embedded. The pitch of the propeller was designed to be 20° at the initial state as indicated in Fig. 14a,c. Application of electric current to the twisted SMA wires rotated each propeller so that the pitch increased (we applied 1.5 A of step current for 2 s). Temporal responses of each propeller shown in Fig. 14c indicated that the variable pitch could be controlled from 20° to 81°. The corresponding movie taken during the experiment is provided as Supporting Movie S6.

**Figure 15:**
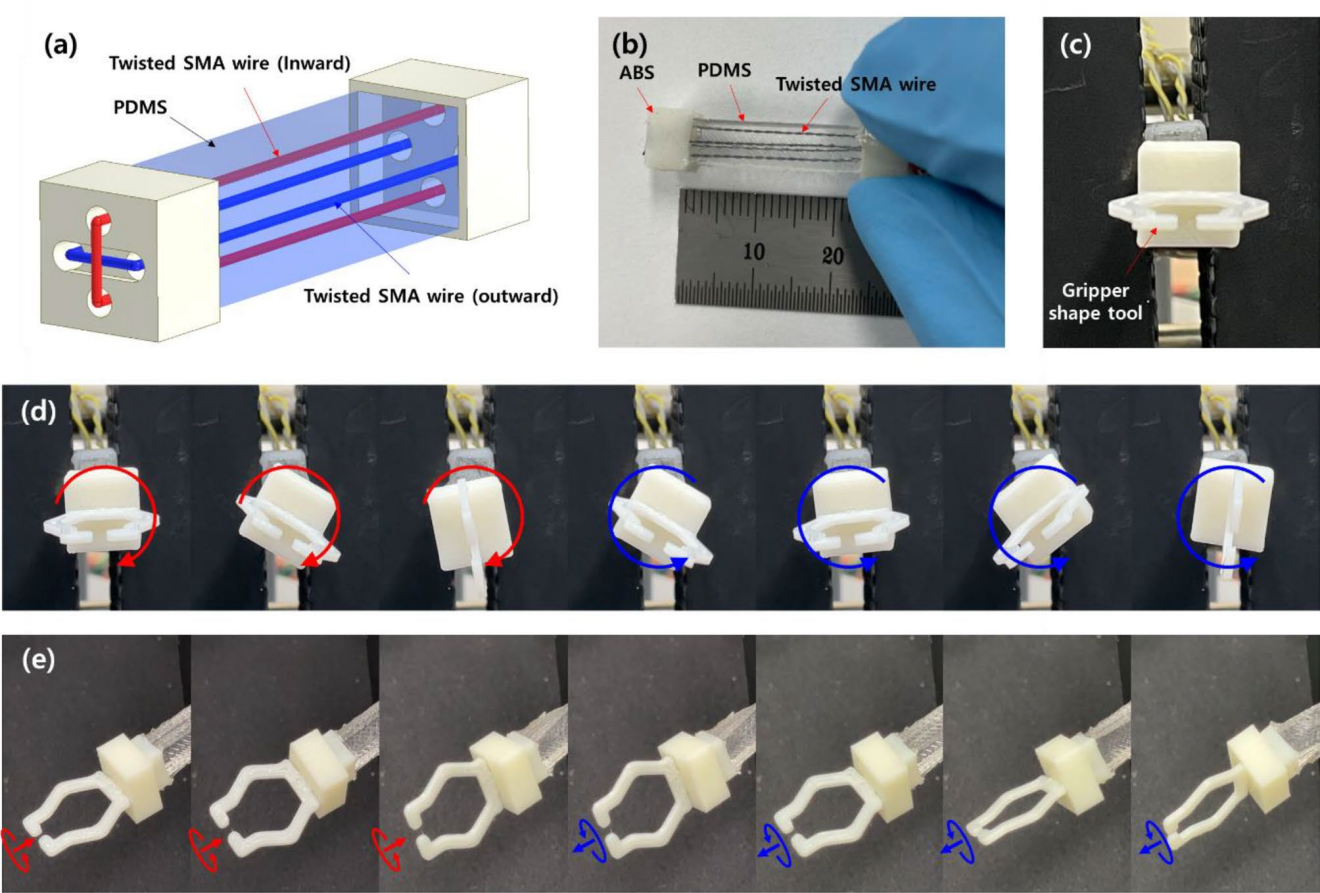
(**a**) Schematic illustrations and (**b**) photo of bi-directional torsional soft actuator. (**c**) A gripper shape tool is attached at the end of the actuator. (**d**, **e**) Snapshots of the bi-directional torsional soft actuator. The outward twisted SMA wire was actuated first approximately for 2 s while the current to the inward twisted SMA wire was zero, and then the inward twisted SMA wire was actuated for approximately 2 s while current to the outward twisted SMA wire was zero.

Regarding many soft robots or morphing structures generally require complicated motions with large deformation, the rotary motion generated by the proposed torsional soft actuator can contribute to the increase of design flexibility, motion complexity and degree of freedom for such applications which need high flexibility and large deformation. Additionally, since our soft actuator can easily be extended for generating bi-directional torsional or rotary motion, it is advantageous for such robotic applications. In this regard, a bi-directional torsional soft actuator prototype was prepared by embedding the outward and inward twisted SMA wires into the PDMS with square cross Section (5 mm × 5 mm and length of 20 mm). Schematic illustration and photo of the fabricated prototype is shown in Fig. 15a,b. A gripper-like tool was installed at the end of the prototype as in Fig. 15c. We captured temporal responses of the bi-directional soft actuator under excitations of each oppositely twisted SMA wire (outward and inward) with 1 A of current, and the results are presented in Fig. 15d,e. We provide the corresponding movie taken during the experiments as Supporting Movie S7. The bi-directional soft actuator generated reliable rotary motion about the actuator center, and it would be useful for adding degree of freedom to soft robotic applications by combining it with the conventional linear movements of many soft actuators. Authors believe that the conceptual demonstrations provided in the present work proves the enough potentials of the proposed soft actuator for versatile applications.

## Conclusions

We present a largely deformable torsional soft morphing actuator based on twisted SMA wires embedded in a PDMS matrix. The design and fabrication procedures were presented, along with the working mechanism of the proposed soft morphing actuator. The actuation performances of the soft actuators were evaluated in terms of their temporal responses and the maximum achievable torsional deformations at various applied currents. The maximum torsional deformation (*θ*_max_) of the actuator was 120.3° for a 20-mm long and 215.5° for a 40-mm long specimen, respectively, at a step current of 700 mA. This performance is better than the maximum torsional deformations of the soft actuator by a single torsional pre-strained SMA wire embedded PDMS (approximately 42° at 750 mA and 69° at 1.25 A for 70-mm long actuator)^13^, the smart soft composite with multi-mode actuations (approximately 160° at 650 mA for 120-mm long actuator)^5^, and the torsional composite actuator based on the polymer scaffold structure (108.4° at 700 mA for 60-mm long actuator)^6^. Potential applications of the proposed soft morphing actuator were presented in the form of soft morphing wings and airfoils. Further design considerations for the proposed soft actuators were presented with demonstrations of the variable pitch control of a propeller and a bi-directional soft actuator. The results showed that the proposed torsional soft morphing actuator can reliably produce large torsional deformation and had enough potentials for versatile applications. The key contributions of the present work include, the proposal of a new type of torsional soft morphing actuator based on twisted SMA wires; presentation of the detailed design and fabrication processes of the proposed soft morphing actuators; and demonstrations of a new type of soft morphing wing, airfoil, and other related applications using the proposed soft actuator. Our work will advance the design of soft actuators, soft robotics, and other related scientific and engineering applications.

## Materials

SMA wire (Flexinol^®^ SMA wire; diameter: 100 and 150 µm; phase transition temperature: 70 °C; Dynalloy, Inc, Irvine, CA, USA.) were used as purchased. The soft matrix was prepared using PDMS (Sylgard™ 184; Dow Corning, Midland, MI, USA) and a curing agent; the mass ratio of the PDMS to the curing agent was 20:1 for all specimens. All specimens were cured at room temperature for 24 h. See Tables 3 and 4 for material properties of the SMA wire and the PDMS.

**Table 3:** Material properties of Flexinol^®^ SMA wire^30^.

Parameter	Value
Austenite start temperature (°C)	68
Austenite finish temperature (°C)	78
Martensite start temperature (°C)	52
Martensite finish temperature (°C)	42

**Table 4:** Material properties of PDMS^30^.

Parameter	Value
Young’s modulus at 25 °C (MPa)	1.8
Tensile strength (MPa)	6.7
Thermal conductivity (W/m–K)	0.27
Temperature range (°C)	–45 to + 200

### Supplementary Information

Supplementary Legends.

Supplementary Movie S1.

Supplementary Movie S2.

Supplementary Movie S3.

Supplementary Movie S4.

Supplementary Movie S5.

Supplementary Movie S6.

Supplementary Movie S7.

## Data Availability

All data generated and analyzed for this study are included in this manuscript.
