# Supplementary material for: Largely deformable torsional soft morphing actuator created by twisted shape memory alloy wire and its application to a soft morphing wing
Source: Sci Rep. 2023 Oct 17;13:17629. doi: 10.1038/s41598-023-44936-4 (PMC10582161; doi:10.1038/s41598-023-44936-4)
Supplement: Supplementary file 1 — Supplementary Legends. [file 41598_2023_44936_MOESM1_ESM.docx]

Largely deformable torsional soft morphing actuator created by twisted shape memory alloy wire and its application to a soft morphing wing

Su-Yeon Lee^1^, Gil-Yong Lee^1,2,*^

^1^ Department of Aeronautics, Mechanical and Electronic Convergence Engineering, Kumoh National Institute of Technology, Gumi, Gyeongbuk 39177, Republic of Korea

^2^ Department of Mechanical Engineering, Kumoh National Institute of Technology, Gumi, Gyeongbuk 39177, Republic of Korea

^*^Corresponding author email: gylee@kumoh.ac.kr

**Supplementary materials**

**Supporting Movie S1.** Motions of the inward and outward twisted SMA wires (without the PDMS) at a 700-mA input current.

**Supporting Movie S2.** Temporal responses of the represented actuator specimens (S1)_out_, (S1)_in_, (S2)_out_, (S3)_out_, (S4)_out_, and (S5)_out_, with a 700-mA input current.

**Supporting Movie S3.** Response of the first morphing wing prototype (Wing out-out) actuated under various applied currents.

**Supporting Movie S4.** Response of the second morphing wing prototype (Wing out-in) actuated under various applied currents.

**Supporting Movie S5.** Response of the soft morphing airfoils.

**Supporting Movie S6.** Response of the propeller prototype.

**Supporting Movie S7.** Response of the bi-directional torsional soft actuator.
